# CROSS-VALIDATION OF THE LICHTENBERG FINANCIAL DECISION RATING SCALE

**DOI:** 10.1093/geroni/igac059.2062

**Published:** 2022-12-20

**Authors:** Emily Flores, Peter Lichtenberg

**Affiliations:** Wayne State University, Detroit, Michigan, United States; Wayne State University, Detroit, Michigan, United States

## Abstract

This study examines the cross-validation of the long form of the Lichtenberg Financial Decision Rating Scale in relation to decision making abilities and suspected financial exploitation.Ninety-five older adult community participants underwent an assessment session which included the LFDRS, cognitive tests and the Independent Living Scale financial management subtest. The LFDRS has four subscales including an intellectual factor that measures choice, understanding, appreciation, and the rationale of decisions. Demographic information was also collected.The concurrent validity of the subscales and LFDRS were examined by Pearson correlations in order to be able to examine the relationship between cognitive test scores, ILS money management scores and LFDRS risk scores. Hierarchical regression analysis was conducted to determine whether cognitive and financial management tests were contributed to the prediction or risk scores greater than demographic variables. LFDRS total was significantly related to executive functioning and female gender. The Financial Situational Awareness subscale showed significant correlations with Trailmaking B but was unrelated to demographics. The relationship of executive functioning to vulnerability to exploitation was largely driven by the Financial Situational Awareness subscale. This finding is consistent with previous studies of the LFDRS and adds to the evidence supporting the concurrent validity of the LFDRS with cognitive functioning.

